# H3N2 canine influenza virus and *Enterococcus faecalis* coinfection in dogs in China

**DOI:** 10.1186/s12917-019-1832-x

**Published:** 2019-04-11

**Authors:** Liwei Zhou, Haoran Sun, Shikai Song, Jinhua Liu, Zhaofei Xia, Yipeng Sun, Yanli Lyu

**Affiliations:** 1 College of Veterinary Medicine, China Agricultural University, No.2 Yuanmingyuan West Road, Beijing, 100193 China; 2 Key Laboratory of Animal Epidemiology of the Ministry of Agriculture and State Key Laboratory of Agrobiotechnology, College of Veterinary Medicine, China Agricultural University, No.2 Yuanmingyuan West Road, Beijing, 100193 China

**Keywords:** Canine influenza virus, H3N2, *Enterococcus faecalis*, Coinfection

## Abstract

**Background:**

In May 2017, 17 dogs in a German Shepherd breeding kennel in northern China developed respiratory clinical signs. The owner treated the dogs with an intravenous injection of Shuang-Huang-lian, a traditional Chinese medicine, and azithromycin. The respiratory signs improved 3 days post-treatment, however, cysts were observed in the necks of eight dogs, and three of them died in the following 2 days.

**Case presentation:**

Quantitative real-time PCR was used to detect canine influenza virus (CIV). All of the dogs in this kennel were positive and the remaining 14 dogs had seroconverted. Two of the dogs were taken to the China Agricultural University Veterinary Teaching Hospital for further examination. Two strains of influenza virus (A/canine/Beijing/0512–133/2017 and A/canine/Beijing/0512–137/2017) isolated from the nasal swabs of these dogs were sequenced and identified as avian-origin H3N2 CIV. For the two dogs admitted to the hospital, hematology showed mild inflammation and radiograph results indicated pneumonia. Cyst fluid was plated for bacterial culture and bacterial 16 s rRNA gene PCR was performed, followed by Sanger sequencing. The results indicated an *Enterococcus faecalis* infection. Antimicrobial susceptibility tests were performed and dogs were treated with enrofloxacin. All 14 remaining dogs recovered within 16 days.

**Conclusions:**

Coinfection of H3N2 CIV and *Enterococcus faecalis* was detected in dogs, which has not been reported previously. Our results highlight that CIV infection might promote the secondary infection of opportunistic bacteria and cause more severe and complicated clinical outcomes.

## Background

Canine influenza virus (CIV) causes acute respiratory infection in dogs [1]. CIV of different origins and subtypes can infect dogs, however, two major subtypes, equine-origin H3N8 and avian-origin H3N2 CIVs, have established stable lineage in canine population. Avian H3N2 CIV was first identified in dogs in southern China in 2006 [2], and the following year, three H3N2 strains were isolated from dogs with severe respiratory disease in Korea [3]. Since then, H3N2 CIVs have been isolated from nasal swabs of dogs experiencing respiratory clinical signs in several regions of China and South Korea [4–8]. In addition, H3N2 CIV has been transmitted to the United States, and was first isolated in the February–March 2015 outbreak in Chicago [9].

CIV infections are usually associated with upper respiratory tract clinical signs, including coughing and rhinorrhea. Severe disease, such as high fever, pneumonia or bronchopneumonia, and death have occasionally been reported [10]. Natural infections of CIV, especially in kenneled dogs, are likely to be associated with other respiratory pathogens, such as canine distemper virus (CDV), canine adenovirus type 2 (CAdV type 2), or canine parainfluenza virus (CPIV), which may increase the severity of disease [11].

*Enterococcus faecalis* is a Gram positive, non-spore-forming, facultative anaerobic bacterium, inhabiting the gastrointestinal tract of humans and animals, and also widely distributed in the environment [12, 13]. *E. faecalis* does not cause disease in healthy humans or animals, despite the nosocomial pathogenicity of *Enterococci* [14]. In dogs, there have only been a few reports of disease caused by *E. faecalis*, but the bacteria have been isolated from cases of urinary tract infections [15–17], periodontitis [18] and endocarditis [19].

CIV coinfection with respiratory bacterial pathogens may increase the pathogenicity [20]. Here, we report coinfection of CIV and *E. faecalis* in dogs for the first time. This case study emphasizes the importance of timely detection and effective treatment of CIV, to reduce the risk of secondary infections and improve outcomes.

## Case presentation

In May 2017, all 17 dogs (aged 2–18 months old) in a German Shepherd breeding kennel in Beijing, developed coughing and rhinorrhea about 4 days after the introduction of a new dog. The breeder administered intravenous Shuang-Huang-lian (60 mg/kg/day) and azithromycin (10 mg/kg/day). Shuang-Huang-Lian, a traditional Chinese medicine formulation comprising alcohol-water extracts of three herbs (Lonicerae Japonicae Flos, Scutellariae Radix, and Fructus Forsythiae), is widely used in China to treat respiratory infection as antimicrobial agents [21, 22]. Respiratory signs reduced 3 days post-treatment, however, cysts of various sizes (ranging from 5 to 10 cm in diameter), were observed by breeder in the ventral neck of eight dogs, and three of them died in the following 2 days. Two dogs with cysts and respiratory clinical signs (dog No.1 and No.2) were taken to the China Agricultural University Veterinary Teaching Hospital (CAUVTH) for examination.

**Fig. 1:**
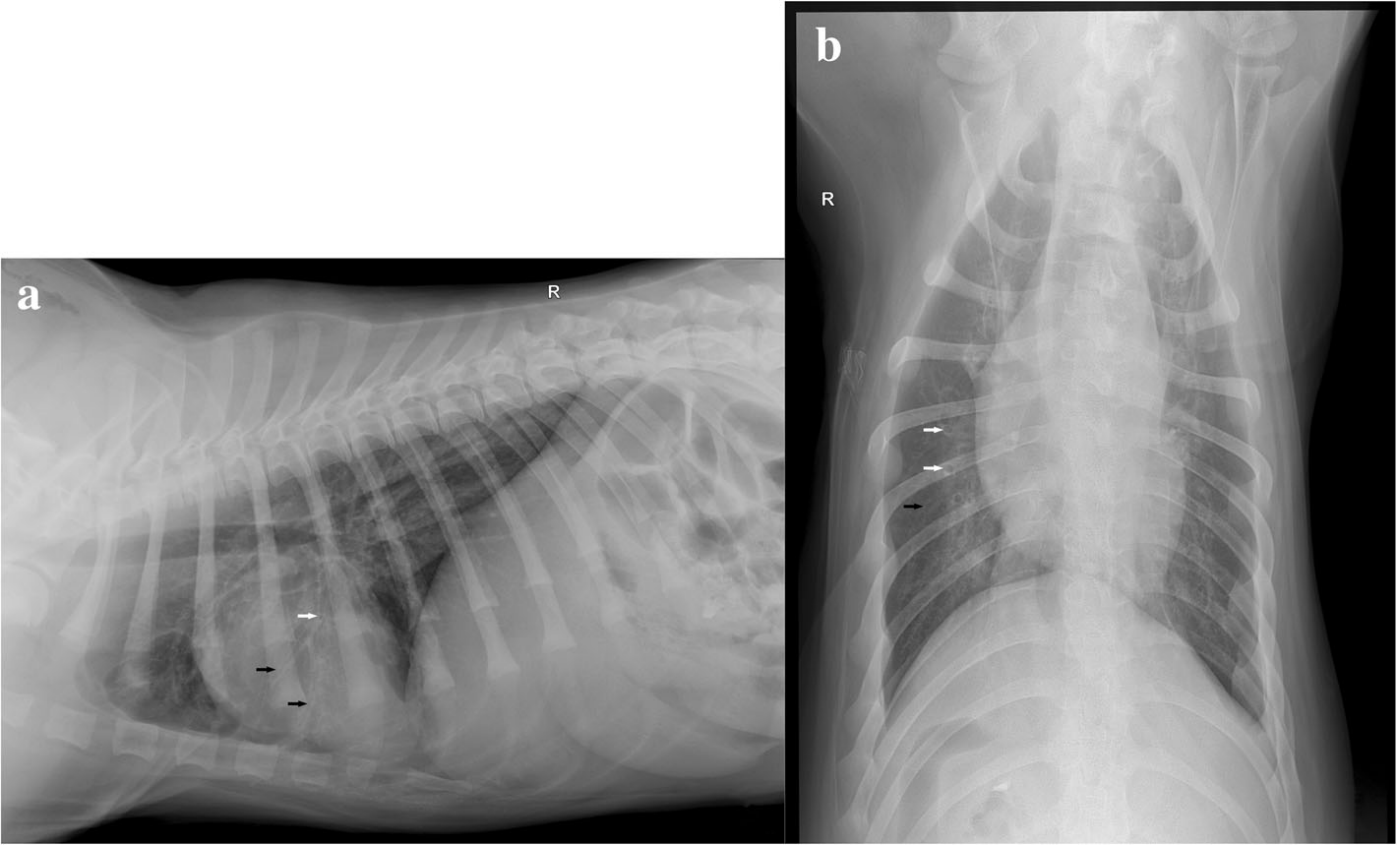
Lateral (**a**) and ventrodorsal (**b**) radiographs of the thorax of dog No. 1. There is a typical bronchial pattern evidenced by tram lines (black arrow) and ring shadows (white arrow), as well as a mild increase in interstitial opacity (unstructured interstitial pattern)

Several diagnostic tests, including a general clinical examination, hematology and serum biochemistry, were performed for the two dogs (Table 1). Hematology showed mild increase in leukocyte, which indicated the animals had inflammation (Table 1). Thoracic radiographs revealed pneumonia (Fig. 1).

**Table 1:** Clinical data of dog No.1 and No.2 in this report

Dog ID	Species, age (months), sex	General examination	Hamaetology			Serum chemistry
			Index	Result	Reference interval	
1	German Shepherd dog, 4, female	38.9°C, rough breathing sounds, a cyst in the neck (10 cm in diameter)	HCT	35.5%	37.3–61.7	Normal
			MCV	57.3 fL	61.6–73.5	
			WBC	17.01 × 10^9/L	5.05–16.76	
			LYM	5.18 × 10^9/L	1.05–5.10	
			MONO	6.96 × 10^9/L	0.16–1.12	
2	German Shepherd dog, 4, male	38.8°C, rough breathing sounds, a cyst in the neck (5 cm in diameter)	RBC	5.53 × 10^12/L	5.65–8.87	Normal
			HCT	33.7%	37.3–61.7	
			MCV	60.9 fL	61.6–73.5	
			WBC	16.93 × 10^9/L	5.05–16.76	
			MONO	1.49 × 10^9/L	0.16–1.12	

Dog No.1 and No.2 were admitted to the China Agricultural University Veterinary Teaching Hospital on the 4th day post-onset of respiratory signs.All abnormal results of hematology are in the table

Nasopharyngeal secretions were collected from dog No.1 and No.2 and four different commercial quantitative real-time PCR (qPCR) assays (Beijing Anheal Laboratory Co., Ltd., China) were used for CDV, CAdV type 2, CPIV and CIV detection. The samples were CIV-positive, but negative for CDV, CPIV and CAdV type 2.

**Fig. 2:**
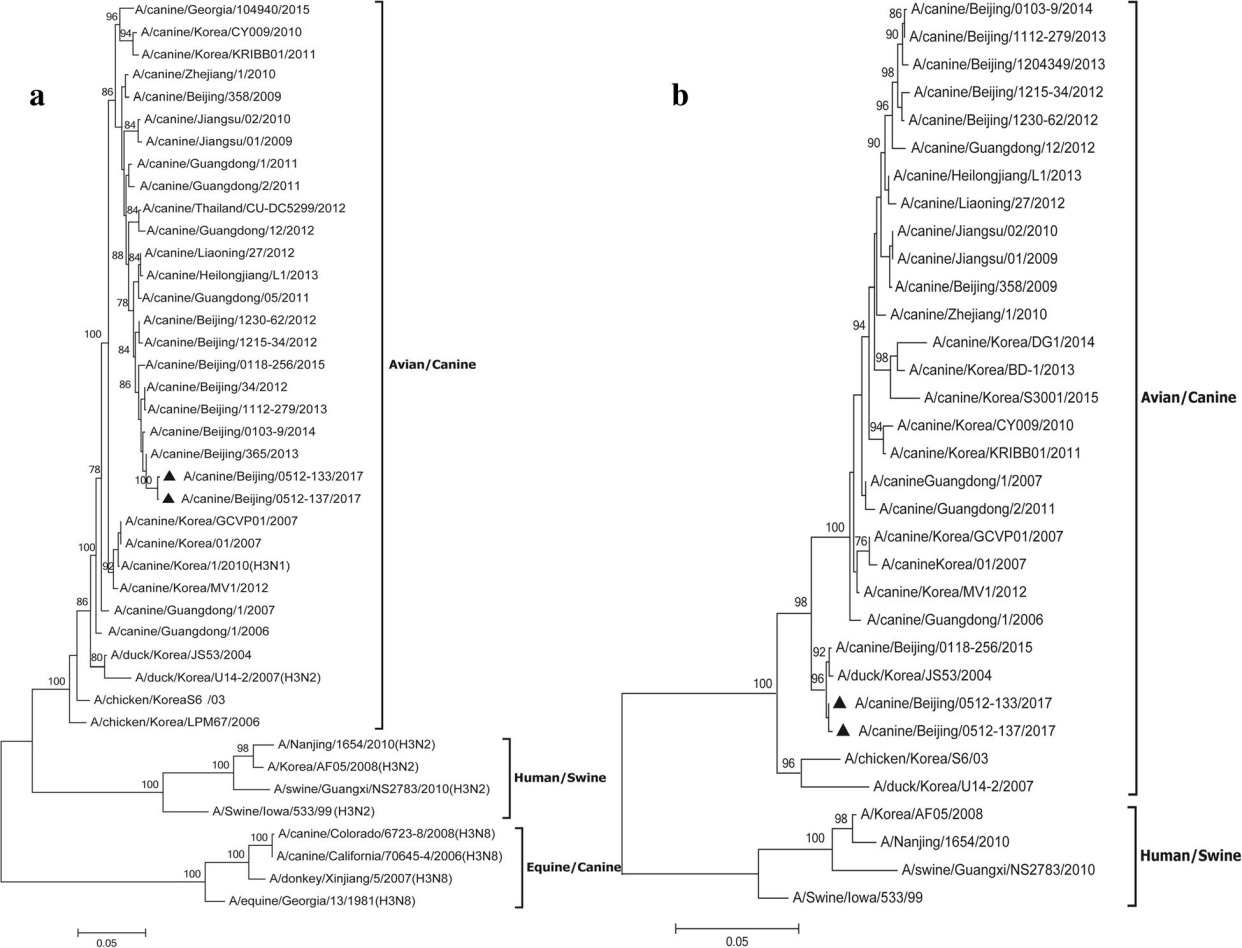
Phylogenetic trees for the **a** HA and **b** NA genes of H3N2 CIVs. Unrooted phylogenetic trees were generated by the maximum likelihood method using Mega 6. The reliability of the tree was assessed using bootstrap analysis with 1000 replicates. Analysis was based on nucleotides 10–1698 of the HA gene and 1–1410 of the NA gene. Virus isolated from dog No.1 and No.2 in the present study are labeled with a triangle. The subtype of viruses that were not H3N2 are shown in parentheses following the virus name. Virus sequences referred to in the tree were obtained from GenBank

Nasopharyngeal secretions from all 12 dogs remaining in the kennel were collected for virus detection as previously described, and all dogs were confirmed CIV-positive. Two samples from dog No.1 and No.2 were inoculated into the allantoic cavity of 9- to 11-day-old embryonated chicken eggs for virus propagation and isolation. Allantoic fluids were harvested after two blind passages and both presented haemagglutinating activity. Subsequently, viral nucleic acid was extracted and the HA and NA genes were amplified by RT-PCR, using universal primers for influenza A virus [23]. Phylogenetic analysis of HA and NA genes clearly demonstrated a close genetic relationship between the two isolates (A/canine/Beijing/0512–133/2017 and A/canine/Beijing/0512–137/2017) and were both avian-origin canine H3N2 (Fig. 2).

Serum samples were collected from 14 remaining dogs on the 4th day and 2 weeks post-onset of respiratory signs, respectively. Hemagglutination inhibition (HI) tests were undertaken using the A/canine/Beijing/0512–137/2017 strain, and the samples collected 2 weeks post-onset were antibody-positive, while samples from day 4 were all negative, indicating seroconversion.

**Fig. 3:**
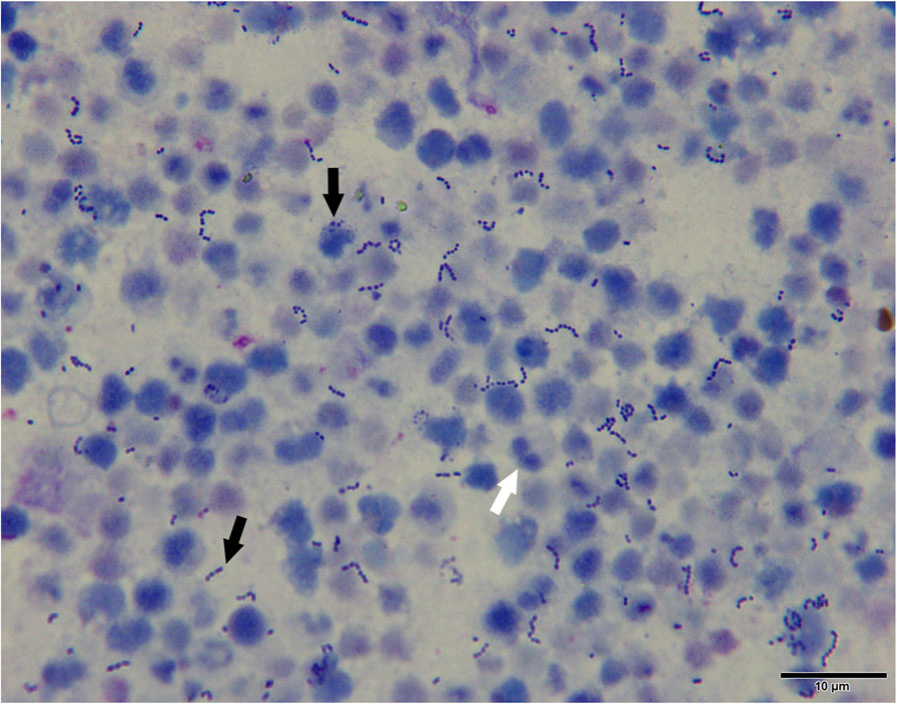
Cytological examination of the cyst fluid from dog No.1. Large numbers of degenerative neutrophils (short arrow), with numerous intracellular and extracellular cocci (long arrow). Wright & Giemsa

Dog No.1 was selected from 8 dogs with cysts for more investigation. Cyst fluid from dog No.1 was sampled using a fine needle aspiration. Cytological examination identified suppurative inflammation associated with numerous cocci (Fig. 3). Subsequently, 20 μL of cyst fluid was plated on sheep blood agar plates for bacterial culture. After incubation at 37 °C for 24 h, non-hemolytic small colonies (0.5–1 mm in diameter), appeared on the plates. To identify the species, bacteria from a single colony was cultured and whole genome DNA was extracted for PCR amplification of the 16 s rRNA gene [24]. Sequencing of the 16 s rRNA gene identified *E. faecalis*. Furthermore, antimicrobial susceptibility tests were carried out to provide guidance for clinical medication, and the results showed multidrug resistance, with sensitivity to enrofloxacin and norfloxacin (Table 2).

**Table 2:** Antimicrobial susceptibility test of clinical *E. faecalis* isolate from dog No.1

Antimicrobial agents	Result
Penicillin	Resistant
Ampicillin	Resistant
Cefalexin	Resistant
Ceftriaxone	Resistant
Vancomycin	Resistant
Erythromycin	Resistant
Tetracyclines	Resistant
Chloramphenicol	Resistant
Florfenicol	Intermediate
Enrofloxacin	Sensitive
Norfloxacin	Sensitive
Clindamycin	Resistant
Amikacin	Intermediate
Doxycycline	Resistant

Treatment with subcutaneous 5% enrofloxacin (10 mg/kg/day), was administered for 5 days. Eight dogs without cysts recovered 1 week post-onset of respiratory signs, and a reduction in cyst size was observed in the remaining five dogs. All dogs recovered within 11 to 16 days of treatment.

## Discussion and conclusion

Influenza A virus infection can cause respiratory symptoms in humans and many animals. Secondary bacterial infections, which are a common complication of influenza virus infection, may significantly increase the severity of the disease and result in poorer outcomes. In humans, *Streptococcus pneumoniae*, *Staphylococcus aureus* and *Haemophilus influenzae* are the three most frequently reported bacteria secondary to influenza infection. Other less common bacteria include *Nocardia* [25], *Mycoplasma pneumoniae* [26], *Mycobacterium tuberculosis* [27], *Legionella pneumophila* [28], and *Campylobacter jejuni* [29]*.* Choi et al. performed a retrospective analysis of 636 swine influenza virus (SIV) cases in pigs and found that *Pasteurella multocida* and *Mycoplasma hyopneumoniae* were the most common bacteria associated with SIV [30]. In addition, artificial infection tests demonstrated that coinfection of SIV with *Haemophilus parasuis* or *Bordetella bronchiseptica* aggravated lung injury in pigs [31]. In birds infected with influenza virus, *Mycoplasma gallisepticum*, *Escherichia coli*, *Riemerella anatipestifer*, *Pasteurella multocida* and other common bacteria have been detected [32, 33]. In dogs, however, apart from one study that isolated *Staphylococcus pseudointermedius* and *Mycoplasma* from the lungs of H3N2 CIV-infected dogs [34], few other bacterial coinfections have been reported.

In this case report, we describe a CIV outbreak in a breeding kennel in northern China. This is the first time that CIV coinfection with *E. faecalis* in dogs has been reported worldwide. In our case, all 17 dogs in the kennel were infected by CIV after the introduction of a new dog. Though the new dog showed no observable clinical signs when it was introduced into the kennel, it was among the first few dogs that showed respiratory symptoms. Previous study showed that clinically healthy dogs can carry respiratory pathogens and could act as sources of infection for susceptible dogs [35]. Therefore, the new dog might be the source of CIV infection. Additionally, 8 of the infected dogs developed cysts. We observed numerous cocci with similar morphology from cyst fluid using cytological examination, and cyst fluid was cultured and 16 s rRNA sequencing was performed. Then, *E. faecalis* was successfully identified, therefore, the dogs were treated with enrofloxacin and cyst sizes reduced. 16 s rRNA sequencing is a cost-effective and efficient method to identify the species of bacteria, however, direct detection of the cyst fluid using metagenomics could be more accurate and comprehensive in identifying the pathogen that co-infected the dogs with CIV. In general, CIV infections are self-limiting, with high morbidity and low mortality. Animal experiments showed that the mortality rate of H3N2 CIV infection is low [3, 7]. In our case report however, three dogs died from coinfection of CIV and *E. faecalis*, therefore we hypothesize that *E. faecalis* infection increased the severity of the disease.

The increased risk of secondary bacterial infections in patients with influenza virus infection may be associated with a variety of factors. For example, an inflammatory response to viral infection may up-regulate expression of molecules that bacteria utilize as receptors, like platelet activating factor receptor can be served as attachment molecule for *S. pneumoniae*, one of the pathogens complicating influenza infection [36]. In addition, virus infection causes sustained desensitization to bacterial toll-like receptor ligands, affecting the normal bacterial clearance mechanism [37]. In our case report, the *E. faecalis* was similar to the *Staphylococcus pseudointermedius* [20] cited in other research as common commensal bacteria in dogs, which could cause opportunistic infections. There is no evidence that CIV infection increases the risk of *E. faecalis* infection in dogs, however, one study found that mice experimentally infected with H3N2 CIV, followed by *Staphylococcus pseudointermedius* 72 h later, resulted in increased bacterial colonization [20]. Meanwhile, studies have shown that immunosuppression enhanced *E. faecalis* colonization [38]. According to the breeder of the dogs in our case report, this was not the first time that he administered the same medical management for dogs experiencing respiratory clinical signs, however, no similar infection had occurred previously. We speculate that CIV infection affects the normal immune mechanism in dogs, making it more susceptible to opportunistic infections, such as *E. faecalis*. Therefore, in addition to symptomatic treatment, we recommend the use of broad-spectrum antibiotics in dogs with CIV infection, to control other possible infections.

Currently, CIV vaccines are rarely used in China, which has caused difficulties in preventing and controlling the epidemic of canine influenza. We emphasize that once dogs develop signs of upper respiratory disease, they should be tested for the presence of CIV infection and be quarantined from other susceptible dogs. In the meantime, since coinfection of CIV with bacteria may affect pathogenicity and disease progression, it is of great importance to recognize secondary bacterial infection as a major clinical complication of influenza infection during disease assessment.

We described a CIV outbreak in a breeding kennel and have confirmed H3N2 CIV and *E. faecalis* co-infection. CIV and *E. faecalis* co-infected dogs had more severe consequences and longer duration compared with those with CIV infection, suggesting CIV infection might promote the secondary infection of opportunistic bacteria and cause more severe and complicated clinical outcomes. This emphasizes the importance of preventing bacterial exposure and improving health care during CIV infection.

## Abbreviations

CAdV: Canine adenovirus
CAUVTH: China Agricultural University Veterinary Teaching Hospital
CDV: Canine distemper virus
CIV: Canine influenza virus
CPIV: Canine parainfluenza virus
PCR: Polymerase chain reactions
qPCR: quantitative real-time PCR
SIV: Swine influenza virus
